# Assessment of the Relationship between Diabetic Retinopathy and Nailfold Capillaries in Type 2 Diabetics with a Noninvasive Method: Nailfold Videocapillaroscopy

**DOI:** 10.1155/2016/7592402

**Published:** 2016-12-13

**Authors:** Seyit Uyar, Ayşe Balkarlı, Muhammet Kazım Erol, Bayram Yeşil, Abdullah Tokuç, Doğan Durmaz, Süheyla Görar, Ayhan Hilmi Çekin

**Affiliations:** ^1^University of Health Sciences Antalya Training and Research Hospital, Department of Internal Medicine, Antalya, Turkey; ^2^University of Health Sciences Antalya Training and Research Hospital, Department of Rheumatology, Antalya, Turkey; ^3^University of Health Sciences Antalya Training and Research Hospital, Department of Ophthalmology, Antalya, Turkey; ^4^University of Health Sciences Antalya Training and Research Hospital, Department of Endocrinology, Antalya, Turkey

## Abstract

*Background and Objectives*. Nailfold capillaroscopy is an easy and noninvasive technique used to investigate dermal microvasculature. Traditional investigations of vascularity do not detect changes until they are well-established in type 2 diabetics. The objective of the current study was to evaluate nailfold capillaries in type 2 diabetes mellitus patients and to determine the association of retinopathy with changes in the nailfold capillaries.* Materials and Methods*. Capillaroscopic findings by nailfold capillaroscopy and fundoscopic examinations were assessed in 216 patients with type 2 diabetes mellitus and 101 healthy controls included in this prospective study.* Results*. Retinopathy was detected in 43.05% of diabetic patients (*n* = 93). Capillaroscopic findings including tortuosity (*p* < 0.001), bushy capillary (*p* < 0.001), neoformation (*p* < 0.001), bizarre capillary (*p* < 0.001), microhemorrhage (*p* = 0.001), capillary ectasia (*p* = 0.002), and aneurysm (*p* = 0.004) were significantly higher in diabetic group than control group. In logistic regression analysis, only tortuosity was shown significant (OR, 2.16; *p* = 0.036). There was also a significant relation between diabetes duration and most of the capillaroscopic findings.* Conclusion*. Capillaroscopic changes were found to be correlated with diabetic retinopathy, in particular with longer disease duration in our study. Capillaroscopic imaging could be a useful new technique for assessment of diabetic microvascular changes.

## 1. Introduction

Diabetes is a complex, chronic illness requiring continuous medical care with multifactorial risk-reduction strategies [1]. Diabetic vascular complications are the most common cause of mortality and morbidity worldwide, with numbers of affected individuals steadily increasing [2]. Diabetic retinopathy (DR) is the most common microvascular complication of diabetes and remains a major cause of preventable blindness [3]. Microaneurysms, leukocyte adhesion, and apoptosis of vascular and neuronal cells are the early changes of DR. Capillary degeneration and development of acellular capillaries cause a reduction in capillary perfusion and hypoxia. Consequently, capillary neovascularization occurs and these findings are characteristic of proliferative DR [3]. The majority of patients with DR have no symptoms until an advanced stage and may rapidly deteriorate. Impairment of the mechanical structure of the vessel wall and vascular endothelial function causes vascular dysfunction and this also fuels the pathogenesis of vascular disease in type 1 diabetes mellitus (T1DM) and type 2 diabetes mellitus (T2DM) [4]. Unfortunately, traditional methods do not detect these complications until they are well-established.

Nailfold videocapillaroscopy (NVC) is an easy, noninvasive, safe, and useful diagnostic tool to evaluate the microvascular structure of nailfold. NVC is used to assess disturbances in the skin capillaries of patients with autoimmune connective tissue disorders, especially in systemic sclerosis. NVC enables more accurate measurement and it is possible to store and analyze capillary data [5].

On the basis of this knowledge, the aim of the current study was to assess the nailfold capillaries to evaluate diabetic microvascular involvement, to determine any correlation between nailfold capillaroscopic findings and retinopathy and to search whether these changes have a relationship with duration of diabetes or not in patients with T2DM.

## 2. Materials and Methods

### 2.1. Patients

The study included 216 patients with T2DM and 101 healthy controls in the Internal Medicine Outpatient Clinic of University of Health Sciences Antalya Training and Research Hospital. Exclusion criteria were a history of ocular and retinal disease, Raynaud phenomenon, collagen tissue disease, drug usage affecting fibrinolysis metabolism (such as glucocorticoids and oral contraceptives), smoking, and occupation with a risk of microtrauma (e.g., farmer and gardener). All the patients were examined by an ophthalmologist for DR and by a rheumatologist for capillary assessment with the NVC device. Approval for the study was granted by the Local Ethics Committee and informed consent was obtained from all patients.

### 2.2. Ocular Examination

All subjects underwent a complete ophthalmic examination including best-corrected visual acuity, slip-lamp biomicroscopy, and dilated fundus examination. DR was confirmed by fundus photography (FFA Visucam NM/FA, Carl Zeiss, Germany) and optical coherence tomography imaging (Cirrus HD-OCT Model 5000, Carl Zeiss Meditec Inc., Dublin, CA, USA) and classified according to the ETDRS (Early Treatment of Diabetic Retinopathy Study, September 1, 2006) guidelines.

### 2.3. Capillaroscopic Assessment

After 20 minutes resting at a room temperature of 20–24°C, immersion oil was applied on the nailfold of all participants for better visualization. Capillaroscopy was applied to 8 fingers (excluding the thumbs) of all participants at ×200 magnification with a capillaroscopy device (Videocap, DS MediGroup, Milan, Italy) by a rheumatologist blinded to patient's condition and 4 images (1 × 1 mm in size) from the middle of the nailfold in each finger were evaluated [6]. A total of 32 images were obtained and recorded on the videocapillaroscopy device for each patient.

The nailfold capillary system was assessed for capillary distribution, density, and morphology according to the Maricq criteria modified by Bergman et al. [7]. In the normal nailfold, the distal capillary rows in the dermal papillae have a parallel course to the nail surface and can be seen in their whole length. These distal capillary rows appear as red and hairpin-shaped in healthy individuals [8]. However, characteristic capillaroscopy findings in patients with rheumatic disease are enlarged capillaries, giant capillaries, neoangiogenesis, capillary loss, and/or avascular areas [9].

**Figure 1:**
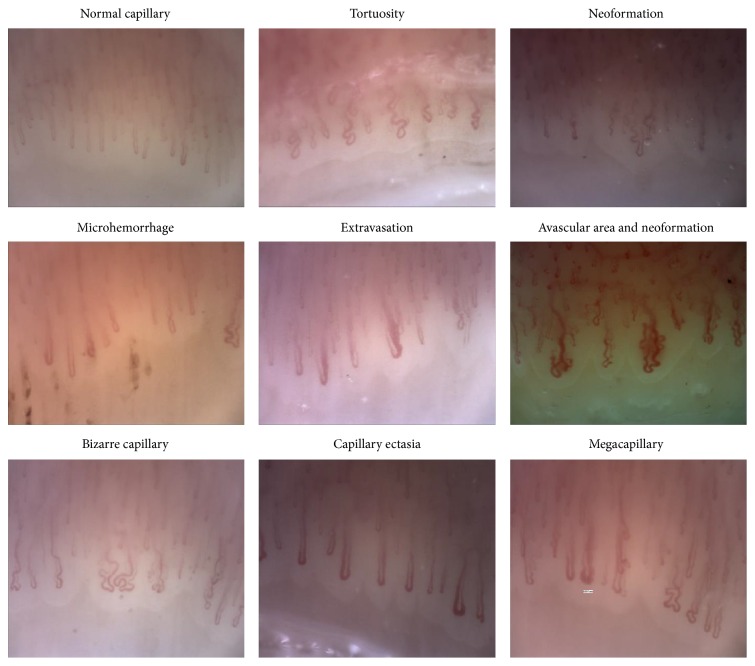
Normal and pathological videocapillaroscopy findings.

Abnormal capillaroscopic findings were defined as follows: (1) tortuosity: 2 or more cross capillaries, each over 1 mm in length; (2) neoangiogenesis: tortuous, bush-like capillaries with marked heterogeneity in size, (a) as the presence of extremely tortuous, bushy, branching, ramified and coiled capillaries, (b) ≥4 capillaries within a single dermal papilla, and (c) thin and branching interconnected capillaries originating from a single loop; (3) microhemorrhage: 2 or more punctate bleeds around a single capillary in at least 2 fingers (separate or confluent microhemorrhage areas); (4) extravasation: leakage of capillary content; (5) avascular area: loss of at least 2 consecutive capillaries or ≤6 capillaries over each 1 mm length; (6) bizarre capillary: capillaries with abnormal appearance but not resembling other defined abnormal capillaries (clover leaf, musical note G, etc.); (7) ectatic capillaries: capillary wall diameter between 0.02 and 0.05 micrometers (regular or irregular); (8) megacapillary: capillary wall diameter >0.05 micrometers (Figure 1) [5, 8].

### 2.4. Statistical Analysis

Descriptive statistics are presented as frequency (*n*), percentage (%), median (minimum–maximum), and mean ± standard deviation (SD). Fisher's exact test and Pearson's chi-square test were used to assess relationships between categorical variables. Conformity to normality of distribution was tested using the Shapiro-Wilks test in groups of sample size ≤ 50 and with the Kolmogorov-Smirnov test in groups of sample size > 50. The difference between two groups was tested using the Mann–Whitney* U* test and Student's* t*-test where appropriate. One-way analysis of variance (one-way ANOVA) with Tukey's HSD post hoc test was used to compare differences between three groups with normal distribution, while the Kruskal-Wallis with Bonferroni-Dunn post hoc test was used for nonnormally distributed data. The Spearman correlation test was applied to test relationships of ordinal or quantitative variables with nonnormal distribution and the Pearson's correlation test to evaluate continuous variables with normal distribution. A value of *p* < 0.05 was considered to be statistically significant. All analyses were performed using SPSS version 22.0.

## 3. Results

### 3.1. Baseline Characteristics

The age was comparable between patients with DR group (*n* = 93; 48 female, 45 male, 62 with proliferative DR, 31 with nonproliferative DR), patients without DR group (*n* = 123; 78 female, 45 male), and healthy control group (*n* = 101; 47 female, 54 male) [60.89 ± 8.281 versus 58.92 ± 8.506 versus 59.41 ± 11.867 years, *p* = 0.316]. Median disease duration of DR positive group was significantly higher than DR negative and control group [14 (0–40) versus 4 (0–25) versus 0 (0) years; *p* < 0.001]. HbA1c levels of patients with DR were higher than patients without DR group [8.7% (5.4–14.6) versus 7.2% (5.0–12.3); *p* < 0.001]. 68.8% of DR positive and 56.9% of DR negative patients have hypertension history and medication (*p* = 0.074) and 22.6% of patients with DR and 35.8% of patients without DR have antilipid treatments (*p* = 0.036) (Table 1).

### 3.2. Frequencies and Comparison of Capillaroscopic Findings of Patients with DR, Patients without DR, and Healthy Control Group

The frequencies of tortuosity [75 (80.6%) versus 71 (57.7%) versus 6 (5.9%); *p* < 0.001], bushy capillary [31 (33.3%) versus 15 (12.2%) versus 0 (0%); *p* < 0.001], neoformation [29 (31.2%) versus 15 (12.2%) versus 0 (0%); *p* < 0.001], bizarre capillary [36 (38.7%) versus 37 (30.1%) versus 7 (6.9%); *p* < 0.001], microhemorrhage [11 (11.8%) versus 5 (4.1%) versus 0 (0%); *p* = 0.001], capillary ectasia [11 (11.8%) versus 7 (5.7%) versus 0 (0%); *p* = 0.002], and aneurysm [10 (10.8%) versus 7 (5.7%) versus 0 (0%); *p* = 0.004] were found significantly increased in diabetics than healthy controls when patients with DR and without DR were compared with healthy controls (Table 1).

### 3.3. Correlation of Capillaroscopic Findings with the Severity of DR

Tortuosity [50 (80.6%) versus 25 (80.6%) versus 71 (57.7%); *p* = 0.002], bushy capillary [25 (40.3%) versus 6 (19.4%) vs. 15 (12.2%); *p* < 0.001], neoformation [22 (35.5%) versus 7 (22.6%) versus 15 (12.2%); *p* = 0.001], and capillary ectasia [10 (16.1%) versus 1 (3.2%) versus 7 (5.7%); *p* = 0.029] were significantly higher in patients with proliferative DR than nonproliferative DR and patients without DR (Table 2).

### 3.4. Comparison of Median Diabetes Years of Patients with and without Significant Capillaroscopic Findings

Median (min–max) diabetes years of tortuosity [10 (0–40) versus 3 (0–23) years; *p* < 0.001], bushy capillary [12.50 (0–40) versus 5 (0–40) years; *p* < 0.001], aneurysm [14 (2–33) versus 6 (0–40) years; *p* = 0.001], neoformation [12 (0–40) versus 5.50 (0–40) years; *p* = 0.002], and bizarre capillary [10 (0–40) versus 5.50 (0–33) years; *p* = 0.049] were significantly higher in diabetic patients who have these findings than patients lacking these findings (Table 3).

### 3.5. Comparison of Median Diabetes Years of Significantly Increased Capillaroscopic Findings between Patients with DR and without DR

Tortuosity [14 (0–40) versus 5.5 (0–25) years; *p* < 0.001], aneurysm [18 (10–33) versus 9 (2–14) years; *p* = 0.003], bizarre capillary [14.5 (0–40) versus 6.5 (0–25) years; *p* < 0.001], and microhemorrhage [16 (8–33) versus 5 (0–9) years; *p* = 0.003] have significantly longer median diabetes years in patients with DR than without DR (Table 3).

### 3.6. Multivariate Logistic Regression Analysis of Significant Capillaroscopic Findings for DR Prediction

Tortuosity was significantly associated with DR [odds ratio 2.106, confidence interval 1.051 to 4.219; *p* = 0.036] (Table 4).

### 3.7. Estimates of Diagnostic Test for Significant Capillaroscopic Findings for DR Detection

**Figure 2:**
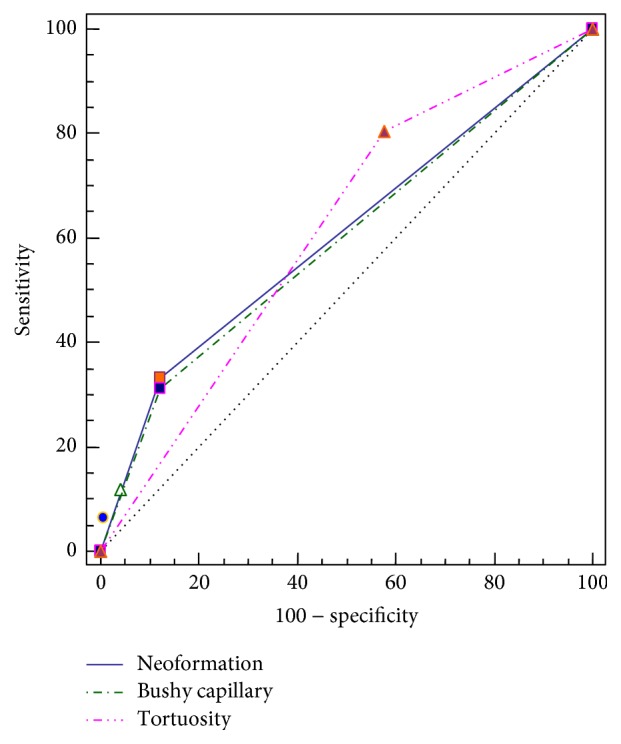
ROC curve of tortuosity, bushy capillary, and neoformation.

The AUC (area under curve) values of tortuosity (0.615), bushy capillary (0.606), and neoformation (0.595) were lower than 80% with ROC (receiver operating characteristic) analysis (Table 5, Figure 2).

## 4. Discussion

DR is the leading cause of blindness and the goal is to detect clinically significant retinopathy before vision is threatened [10]. To identify individuals at risk of DR progression and early intervention can limit vision loss and reduce the costs associated with managing more advanced disease. NVC has been used for the analysis of microvascular structure especially in rheumatic disease and in some extrarheumatic diseases [5]. Any disease affecting the vascular structures may give findings on NVC and there have been many studies assessing capillaroscopic findings in different diseases [11–14]. Therefore, capillaroscopic investigations in DM patients were started in the 1960s [15]. However, there are limited data in literature related to this subject, especially in respect of T2DM due to complex nature of retinopathy [16–18].

In the present study, we demonstrated that tortuosity, bushy capillary, neoformation, bizarre capillary, microhemorrhage, capillary ectasia, and aneurysm were significantly higher in patients with T2DM than healthy controls. Capillaroscopic findings including tortuosity, bushy capillary, neoformation, and capillary ectasia were also significantly higher in patients with proliferative DR than patients with nonproliferative DR and without DR. These findings show that there is a microvascular involvement in T2DM and a strong correlation with DR, and NVC can detect changes in the nailfold capillaries precisely. In 1997, Chang et al. evaluated 35 patients with diabetes (10 without DR, 10 with background DR, and 15 with proliferative DR) and 20 healthy controls. Tortuosity was the highest findings in group of proliferative DR (68%). It was found %20 in control group and %23 in group of without DR [16]. Meyer et al. studied density, diameters, and morphology of nailfold capillaries in 16 patients with T1DM and 19 with T2DM. They found tortuous and dilated capillaries that could indicate microangiopathy by means of NVC [17]. In another study, 49 patients (21 with T1DM and 28 T2DM) and 39 controls were evaluated by Barchetta et al. They performed NVC, ophthalmoscopy, and retinal fluorangiography to all subjects and used quantitative evaluation of NVC and score. They found that increased density, irregular length and distribution of capillary loss, aberrant morphological alterations such as tortuosity, ramifications, and bushes, presence of exudates, oedema, and flux abnormalities were detected by NVC in diabetic patients. Moreover, according to Barchetta et al. study, NVC was capable of identifying alterations in almost %50 of patients with diabetes without retinopathy [18]. Our study is the most comprehensive study in this subject; more patients with T2DM and more healthy controls were evaluated. Furthermore, more and actual capillaroscopic findings were evaluated in this study. These findings can be an indicative of microvascular involvement for further research in T2DM patients.

Diabetes years of patients with tortuosity, bushy capillary, aneurysm, neoformation, and bizarre capillary were longer than diabetic patients lacking these findings regardless of retinopathy in our study. When we compared the median diabetes years of significantly present capillaroscopic findings by the presence of DR, diabetes years of patients having tortuosity, aneurysm, bizarre capillary, and microhemorrhage were significantly longer in patients with DR than patients without DR. Positive correlation of capillaroscopic findings and diabetes duration was also stated in Chang et al. and Meyer et al. studies, whereas Barchetta et al. found that NVC findings were independent from duration of diabetes [16–18]. Although these findings does not predict when the microvascular changes formed, there is a significant correlation between diabetes years and capillaroscopic findings in our study. It can be assumed that early detection of tortuosity, aneurysm, bizarre capillary, and microhemorrhage may be a precursor of DR.

Although tortuosity was the most valuable capillaroscopic finding in logistic regression analysis, it cannot be used as a diagnostic tool since the AUC value was less than 80% in ROC curve (Figure 2).

Compared to previous studies, this cross-sectional study has more T2DM patients and more capillaroscopic findings were evaluated. Our study population was homogeneous in terms of age and hypertension history between groups. HbA1c level was higher in patients with DR as expected and more patients were using antilipid drug in DR negative group. Relationship of other diabetic complications, such as nephropathy and neuropathy, and other comorbidities of patients with NVC findings were not evaluated in this study as a limitation. Also, quantitative evaluation of NVC findings and NVC score were not performed.

## 5. Conclusions

Our data have showed that there is a significant correlation with capillaroscopic findings and DR, and NVC can detect microvascular changes in T2DM patients without clinically apparent retinopathy. These findings may guide the detection of T2DM associated retinopathy and microvascular complications earlier. Capillaroscopic findings including tortuosity, bushy capillary, aneurysm, neoformation, and bizarre capillary were significantly linked with a longer DM duration and DR positive patients in our study. We suppose that tortuosity may be the leading finding for diagnosis of early DR according to our data. The evaluation of nailfold capillaroscopic findings may be a new modality for vascular assessment of diabetic patients to diagnose and follow up microvascular complications. Further NVC studies are needed to determine the timing and relationship of other comorbidities of DM.

## Figures and Tables

**Table 1:** Demographical characteristics and frequencies of capillaroscopic findings of patients with DR, patients without DR, and healthy controls.

	Diabetic patients (*n* = 216)		Controls (*n* = 101)	*p*
	DR (+) (*n* = 93)	DR (−) (*n* = 123)		
Mean age (SD)	60.89 (8.21)	58.92 (8.506)	59.41 (11.867)	0.316
Male gender, *n*	45 (36.6%)	45 (48.4%)	54 (53.5)	**0.033**
Hypertension, *n*	64 (68.8%)	70 (56.9%)	0	0.074
Hyperlipidemia, *n*	21 (22.6%)	44 (35.8%)	0	**0.036**
HbA1c, % (min–max)	8.7 (5.4–14.6)	7.2 (5.0–12.3)	0	**<0.001**
Diabetes years, median (min–max)	14 (0–40)	4 (0–25)	0	**<0.001**
Tortuosity, *n*	75 (80.6%)	71 (57.7%)	6 (5.9%)	**<0.001**
Bushy capillary, *n*	31 (33.3%)	15 (12.2%)	0 (0%)	**<0.001**
Neoformation, *n*	29 (31.2%)	15 (12.2%)	0 (0%)	**<0.001**
Bizarre capillary, *n*	36 (38.7%)	37 (30.1%)	7 (6.9%)	**<0.001**
Microhemorrhage, *n*	11 (11.8%)	5 (4.1%)	0 (0%)	**0.001**
Capillary ectasia, *n*	11 (11.8%)	7 (5.7%)	0 (0%)	**0.002**
Aneurysm, *n*	10 (10.8%)	7 (5.7%)	0 (0%)	**0.004**
Extravasation, *n*	6 (6.5%)	1 (0.8%)	0 (0%)	NA
Megacapillary, *n*	1 (1.1%)	0 (0%)	0 (0%)	NA
Meander capillary, *n*	4 (4.3%)	3 (2.4%)	0 (0%)	NA
Avascular area, *n*	3 (3.2%)	0 (0%)	0 (0%)	NA
Interstitial edema, *n*	1 (1.1%)	1 (0.8%)	0 (0%)	NA

NA: not applied; DR: diabetic retinopathy.

**Table 2:** Correlation of capillaroscopic findings with severity of DR.

	Proliferative DR (*n* = 62)	Nonproliferative DR (*n* = 31)	Patients without DR (*n* = 123)	*p*
Tortuosity, *n*	50 (80.6%)	25 (80.6%)	71 (57.7%)	**0.002**
Bushy capillary, *n*	25 (40.3%)	6 (19.4%)	15 (12.2%)	**<0.001**
Neoformation, *n*	22 (35.5%)	7 (22.6%)	15 (12.2%)	**0.001**
Capillary ectasia, *n*	10 (16.1%)	1 (3.2%)	7 (5.7%)	**0.029**
Bizarre capillary, *n*	25 (40.3%)	11 (35.5%)	37 (30.1%)	0.372
Microhemorrhage, *n*	9 (14.5%)	2 (6.5%)	5 (4.1%)	NA
Aneurysm, *n*	9 (14.5%)	1 (3.2%)	7 (5.7%)	NA
Extravasation, *n*	6 (9.7%)	0 (0%)	1 (0.8%)	NA
Megacapillary, *n*	1 (1.6%)	0 (0%)	0 (0%)	NA
Meander capillary, *n*	4 (6.5%)	0 (0%)	3 (2.4%)	NA
Avascular area, *n*	3 (4.8%)	0 (0%)	0 (0%)	NA
Interstitial edema, *n*	0 (0%)	1 (3.2%)	1 (0.8%)	NA

NA: not applied; DR: diabetic retinopathy.

**Table 3:** Comparison of median diabetes years of patients with and without significant capillaroscopic findings and comparison of median diabetes years of significant capillaroscopic findings patients with DR and without DR.

			Median (min–max) diabetes years		*p* ^*x*^	*p* ^*y*^
			Patients with DR	Patients without DR		
Tortuosity	+	10 (0–40)	14 (0–40)	5.5 (0–25)	**<0.001**	**<0.001**
	−	3 (0–23)				
Bushy capillary	+	12.50 (0–40)	14 (0–40)	8 (0–25)	**<0.001**	0.169
	−	5 (0–40)				
Aneurysm	+	14 (2–33)	18 (10–33)	9 (2–14)	**0.001**	**0.003**
	−	6 (0–40)				
Neoformation	+	12 (0–40)	14 (0–40)	8 (0–25)	**0.002**	0.143
	−	5.50 (0–40)				
Bizarre capillary	+	10 (0–40)	14.5 (0–40)	6.5 (0–25)	**0.049**	**<0.001**
	−	5.50 (0–33)				
Microhemorrhage	+	12.50 (0–33)	16 (8–33)	5 (0–9)	0.050	**0.003**
	−	7 (0–40)				
Capillary ectasia	+	10 (2–27)	10 (2–27)	8 (4–12)	0.203	0.273
	−	7 (0–40)				

(+): capillaroscopic finding is present; (−): capillaroscopic finding is absent. DR: diabetic retinopathy;*p* ^*x*^: comparison of median diabetes years of patients with versus without significant capillaroscopic finding*; p* ^*y*^: comparison of median diabetes years of significant capillaroscopic findings patients with DR versus without DR.

**Table 4:** Multivariate logistic regression analysis of significant capillaroscopic findings.

	*p*	OR (95% CI)
Tortuosity	**0.036**	2.106 (1.051–4.219)
Bushy capillary	0.270	2.754 (0.455–16.648)
Neoformation	0.919	1.098 (0.182–6.635)
Microhemorrhage	0.119	2.505 (0.790–7.941)
Bizarre capillary	0.803	1.086 (0.568–2.077)
Capillary ectasia	0.778	1.172 (0.388–3.545)
Aneurysm	0.592	1.359 (0.443–4.173)

**Table 5:** Estimates of diagnostic test for significant capillaroscopic findings for DR detection.

	AUC	95% CI
Tortuosity	0.615	0.540 to 0.689
Bushy capillary	0.606	0.528 to 0.683
Neoformation	0.595	0.517 to 0.673
Microhemorrhage	0.539	0.460 to 0.617
Bizarre capillary	0.543	0.465 to 0.621
Capillary ectasia	0.531	0.452 to 0.609
Aneurysm	0.525	0.447 to 0.604

AUC: area under curve; CI: confidence interval.
